# Impact Resistance Study of Three-Dimensional Orthogonal Carbon Fibers/BMI Resin Woven Composites

**DOI:** 10.3390/ma13194376

**Published:** 2020-10-01

**Authors:** Yanqi Hu, Zekan He, Haijun Xuan

**Affiliations:** College of Energy Engineering, Zhejiang University, Hangzhou 310027, China; yanqihu@zju.edu.cn (Y.H.); laokan@zju.edu.cn (Z.H.)

**Keywords:** three-dimensional woven composites, carbon fiber, impact resistance, gas gun, multiscale modeling

## Abstract

Three-dimensional woven composites have been reported to have superior fracture toughness, fatigue life and damage tolerance compared with laminated composites due to through-thickness reinforcement. These properties make them lighter replacements for traditional high-strength metals and laminated composites. This paper will present impact resistance research on three-dimensional orthogonal woven composites consisting of carbon fibers/bismaleimide resin (BMI). A series of impact tests were conducted using the gas gun technique with the impacted target of 150 mm × 150 mm × 8 mm (length × width × thickness) and the cylindrical titanium projectile. The projectile velocity ranged from 180 m/s to 280 m/s, generating different results from rebound to perforation. This paper also presents a multiscale modeling strategy to investigate the damage and failure behavior of three-dimensional woven composites. The microscale and mesoscale are identified to consider the fiber/matrix scale and the tow architecture scale respectively. The macroscale model was effective with homogenized feature. Then a combined meso-macroscale model was developed with the interface definitions for component analysis in the explicit dynamic software LS-DYNA. The presented results showed reliable interface connection and can be used to study localized composites damage at a relatively high efficiency.

## 1. Introduction

Three-dimensional (3D) woven composites have attracted the interest of academia and industry thanks to their damage tolerance characteristics and automated fabric manufacturing [1]. There are three different types of yarns, namely, warp yarns, weft yarns and binder yarns. For 3D orthogonal weave composites (3DOWCs), the three types of yarns are placed in three mutually orthogonal directions. Compared with layer structural laminates, the delamination resistance and out-of-plane properties, due to the use of z-binders, enhance the impact performance of such 3D woven composite systems [2]. Hence the practical application of 3D woven composites, such as in the aerospace industry, aims primarily to achieve high impact resistance with relative low weight. Weight saving further will reduce fuel consumption, be more environmentally friendly and 3D contour weaving and preform technique enable a shaped cross section. The LEAP jet engine, for example, applied 3D woven composites in the blades and fan case [3]. It was produced by CFM International (Cincinnati, Oh, USA. a 50/50 joint venture of Snecma and General electric company) and was regarded as the next generation aeroengine serving narrow-body aircraft. According to airworthiness regulations [4], before an engine can be used in commercial service, the performance of the fan containment system must be demonstrated in a full-scale engine fan-blade-out test. However, due to the extremely high cost, ballistic tests are often carried out to prestudy the impact resistance of composites.

Many researchers have conducted impact tests of composites with various textile structures and reinforced fibers/resin combinations. Poutluri et al. [5] studied the damage tolerance of four 3D woven composites at the energy level ranging from 5–30 J and found that the damage resistance of 3D weaves was significantly higher than 2D weaves and UD (unidirectional) cross-ply. Among the 3D weaves, the modified layer-to-layer laminate had the highest damage resistance, followed by layer-to-layer and 3DTAWCs (3D through-thickness angle-interlock woven composites), while the orthogonal laminate had the lowest damage resistance. Bahei-El-Din and Zikry [6] tried to identify the quasistatic perforation failure mechanism in 3DOWCs under displacement rate of 10–80 m/s. At high impact velocities, wave propagation effects were significant and resulted in penetration at the impact face. Luo [7] and Lv [8] et al. conducted impact tests with impact velocities of 20–55 m/s. Luo found that the energy absorption in hybrid 3DOWCs was strain-rate-sensitive. Lv discovered that the failure stress and energy absorption in warp/weft yarns was affected by the corresponding yarns’ linear density. Hao et al. [9] studied the behavior of 3D woven glass fiber composite plate and T-beams subjected to quasistatic indentation (2 mm/min) and impact loadings (17.5–22.5 m/s). They reported higher energy absorption by the T-beam as opposed to the composite plate. Tsai [10] investigate the mechanical properties of CNT (carbon nanotubes)/Cu by nanoindentation tests. They concluded that enhanced mechanical response is dominated by CNT addition rather than grain size reinforcement. Xuan et al. [11] conducted ballistic impact tests of the 2D statin woven and triaxial braided composites at the impact energy level between 250–377 J. The impact resistance capacity of two different 2D composites was compared. Studies of impact damage tolerance of 3D composites have focused on low velocity. Experimental data was insufficient when very high speed and high energy were involved.

The development of numerical methods has helped much to obtain deeper understanding of the behavior of composites, such as strain-rate dependency, material failure/damage, the aging and thermal effect. Fiber-reinforced composites are known as hierarchical materials with three structural levels [12]: the macroscale, which represents the structural scale for analysis; the mesoscale, representing the textile pattern of architecture; and the microscale, containing the fiber and matrix microconstituents. Antin [13] developed a multiscale modeling approach to estimate the effect of defects on the strength on UD carbon fiber composites. Ivanov [14] revealed two stages in damage accumulation: (1) transverse crack initiation; (2) progressive crack density growth and delamination initiation. The research team lead by Binienda [15,16,17,18] has been devoted to study the impact response of triaxial braided composites through both experimental and numerical methods. Li [17] developed a mesoscale analysis model for simulation of straight sided tensile tests. Material failure and progressive damage of the fiber tows and tow interfaces were taken into account in the model to effectively predict the damage and interface debonding. Hu [18] combined macroscale and mesoscale models by an interface linking method, making it possible to combine the advantages of both scales—the high computation efficiency of macroscale model and the modeling sophistication of the mesoscale model. Nie [19] further improved mesomechanical model with a smaller number of elements and fewer degrees of freedom. Sun [20] proposed a novel hybrid element modeling methodology, which combined different scales of finite element modeling into a single analysis. 

This paper aims to evaluate the failure modes and ballistic resistance of a 3DOWCs used in the aeroengine containment fan case. The next section presents a detailed ballistic impact testing program with energy level ranging from 320 to 770 J and the results are then analyzed. Then the finite element analysis is conducted to simulate the ballistic behavior of carbon fibers/BMI resin composites based on the observation data of experimental results. After that, the combined multiscale modeling method is used, which is able to capture the detailed response in the local area and to realize high computing efficiency at the same time. Finally, the results from these studies are summarized and concluded.

## 2. Materials and Experiments

### 2.1. Materials

The material system discussed in this paper is 3DOWCs consisting of Toray T700 carbon fibers and bismaleimide resin, which will be referred as T700 and BMI-A later. The T700 fiber is a high-strength, standard modulus fiber where tensile strength is needed. It is known to behave as linear elastic material exhibiting an abrupt or brittle failure. The BMI resins are of high-performance thermosetting polymers that possess a range of attractive properties for industrial applications, particularly in the aerospace materials sector. Their monomers are molecules that are terminated by two maleimide functional groups, usually containing multiple aromatic moieties in order to enhance the cured properties. Typical properties associated with cured BMI systems often include high (dry) glass transition temperatures (230–380 °C), good hot-wet performance, constant electrical properties and low flammability [21]. The detailed constituent properties are listed in Table 1.

**Figure 1:**
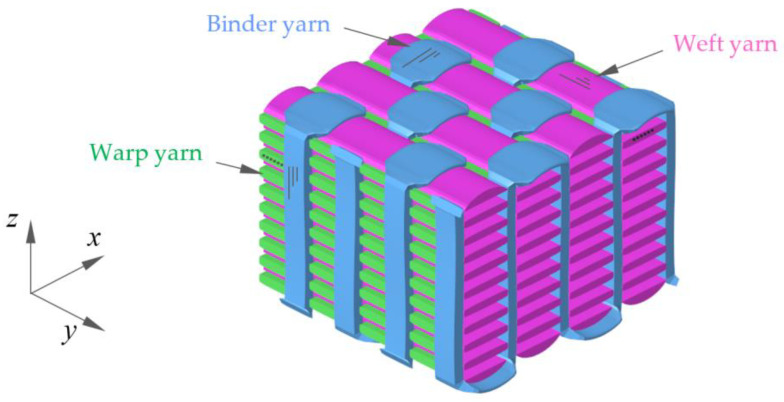
Structure of three-dimensional orthogonal weave composites (3DOWCs; matrix is not shown).

It is widely recognized that composites should be analyzed not only from the material aspect but also from the structure aspect. A typical 3D orthogonal woven architecture consists of three different types of yarns, which are warp yarns, weft yarns and binder yarns, shown in Figure 1. In this study, each yarn consisted 12 k T700 carbon fibers, which amounts to 800 Tex (The Tex standard and uses 1000 m of thread per gram as the starting point. Units = g/km). The materials were woven at Tiangong University and then molded with a resin transfer molding (RTM) process at AECC BIAM (Aero Engine Corporation of China, Beijing Institute of Aeronautical Materials). Before resin injection, the mold along with the weaves were preheated to 103 ± 3 °C. Pressure was stepwise increased to discharge bubbles until injection was completed. The whole device was then preserved at 160 ± 3 °C for 1 h, 180 ± 3 °C for 1 h, 200 ± 3 °C for 2 h, and 250 ± 3 °C for 4 h. Structural parameters are shown in Table 2. The composite panels were in rectangular plate shape and then cut to smaller samples. Total fiber volume fraction was around 54%.

### 2.2. Impact Test

**Figure 2:**
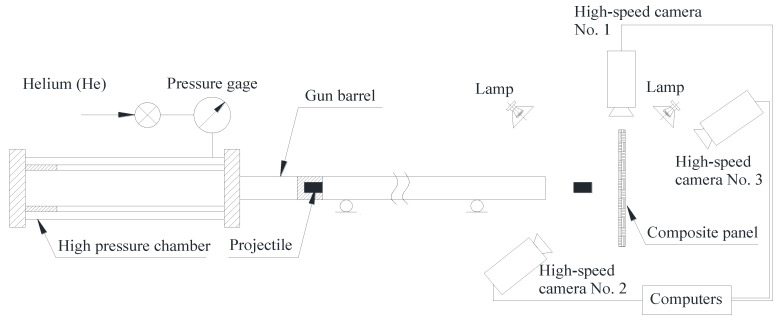
Schematic diagram of the one-stage gas gun.

The ballistic impact tests were conducted using a gas gun at Nanjing University of Aeronautics and Astronautics. Schematic diagram was shown in Figure 2. The gas gun consisted of a barrel, firing valve, and high-pressure chamber. At the target side, there were three high-speed cameras (Phantom V2512) used during the test. Camera No.1 recorded the projectile velocity, including initial impact velocity and residual velocity. Its recording speed was 15 000 fps (frames per second). Cameras No. 2 and 3 were used to capture the panel response from the front and back respectively, with a recording speed of 25,000 fps.

**Figure 3:**
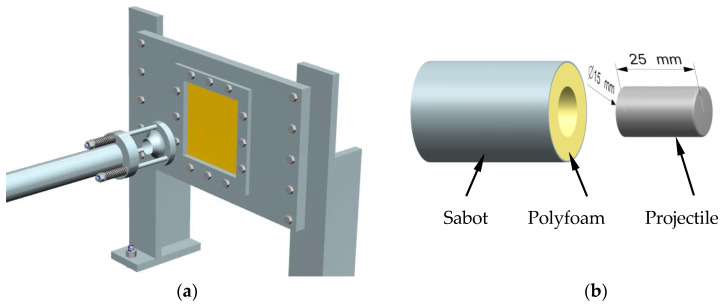
Composite specimen mounting method (**a**) and the projectile dimensions (**b**).

The composite specimen was fixed along four edges by being sandwiched between two rigid steel plates, which were in the shape of a square picture frame, as shown in Figure 3a. The specimen was 150 mm × 150 mm × 8 mm (length × width × thickness) in scale. As there were four fixed edges of 5 mm, the effective impact area was 145 mm × 145 mm × 8 mm (length × width × thickness). The cylindrical projectile was made of Ti-6Al-4V titanium alloy (TC4), weighing 19.7 g, 25 mm long and 15 mm diameter, as shown in Figure 3b. It was mounted in an aluminum alloy sabot full of polyfoam that had a projectile shape inside. During the test, the sabot will be stopped by a conical structure, allowing the projectile to be released and to impact the target.

### 2.3. Test Results

Twelve ballistic impact tests were conducted. The results are summarized in Table 3. The projectile velocity ranged from 180 to 280 m/s (320–774 J), resulting in different composite panel damage and different projectile residual velocity. In Test 8, the projectile was stuck in the composites, in which case the residual velocity was zero and it was believed that all kinetic energy was absorbed by the composites. Therefore, the ballistic limit was around 232 m/s, which amounted to 530 J kinetic energy.

**Figure 4:**
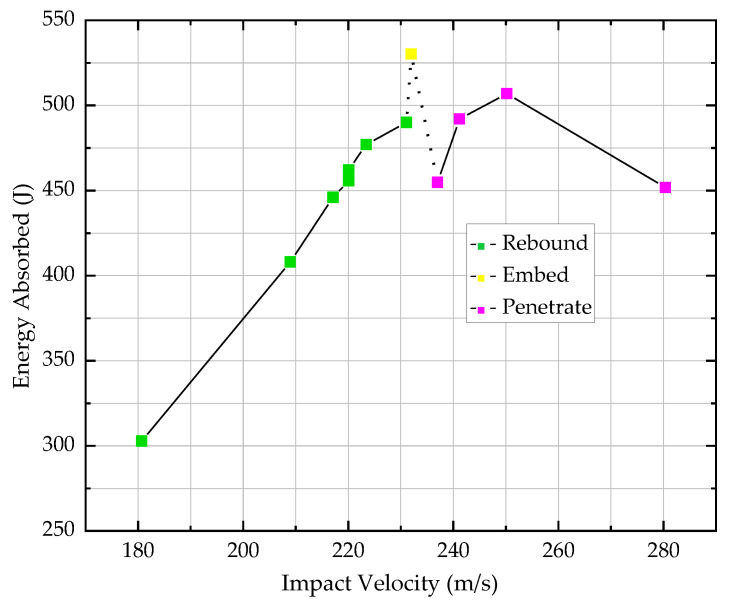
Test results of energy absorbed by composites vs. impact velocity.

Test results were also plotted in Figure 4. It showed that the energy absorption did not change monotonously with the impact velocity. Generally, it increased with the elevation of impact velocity, reached peak value at the ballistic limit and then dropped down, followed by another increase-peak-decrease. The first peak was more critical because test results transformed from rebound to penetration. However, when attention came to the postpeak part, the drop of energy absorption after ballistic limit was noticed, which might be missed if the impact velocity step was large. It might be explained by the large deformation effect. If the deflection of the plane is close to or exceeds its thickness, the in-plane membrane force will be very significant, which will increase the load-bearing capacity [22,23]. In this study, the plane underwent large deflection when the composite target was not penetrated, and much smaller deflection when penetrated. This was found to be consistent with the out-of-plane deformation analysis in Section 3.2.

When the composite target was penetrated, the increase-peak-decrease was a common trend that was similar with report by Ma [24] and Li [25]. The strain rate effect of the material was a dominant factor that allowed the impact energy propagate to a large area of the target at a very high stress wave velocity during a relatively short period of time. Then, as the impact velocity increased, the interaction time between the projectile and the composite target decreased, which led to the energy absorption descending.

**Figure 5:**
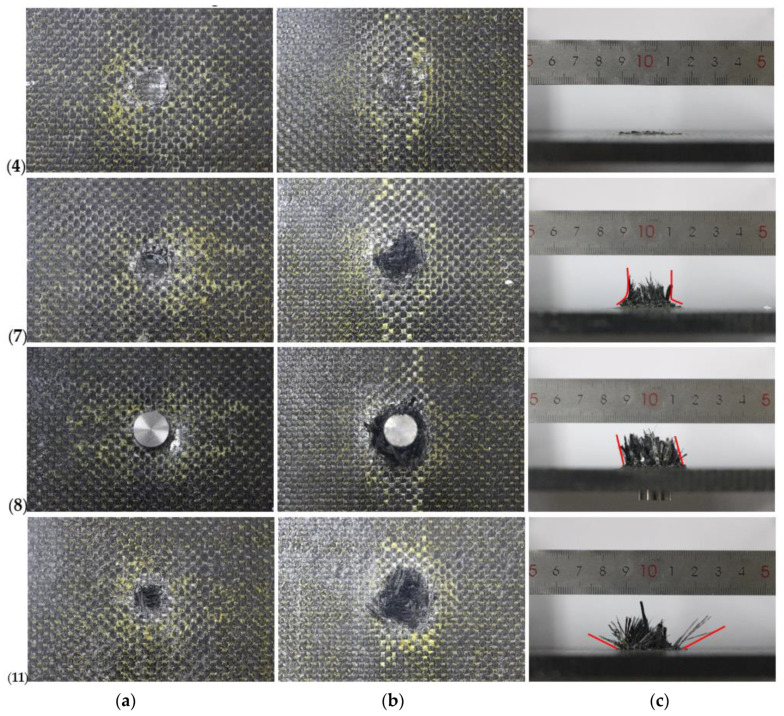
Composite damage of (**a**) front surface (**b**) back surface (**c**) out-of-plane for test 4, 7, 8, 11.

Composite damage is shown in Figure 5. When impact velocity was far lower than the ballistic limit (232 m/s), the projectile failed to penetrate the specimen. It just left a small impact pit on the front (impact) surface and a slight bulge on the back surface, as it is shown in Figure 5 (4, a–c) for test 4 with impact velocity of 220.1 m/s. Only a few fiber breaks can be seen. With the impact velocity increase, the dent became deeper and fiber yarn broke more on the back surface as it happened in Figure 5 (7, a–c), for test 7 with impact velocity of 231.1 m/s. Despite the severe fiber breakage on the back surface, which indicated that more energy can be absorbed, the projectile did not perforate the composites. Until the impact velocity increased as high as the ballistic limit, the fiber yarn broke all the way through the thickness direction, and the projectile was embedded, shown in Figure 5 (8, a–c).

For the specimen impacted by a projectile over 232 m/s, there was a clean circular hole on the front surface, extensive fiber breakage and fiber pull-out on the back surface, as shown in Figure 5 (11, a–c). The clean-cut circular hole shape indicated that the main failure modes on the front surface were fiber shear failure. On the back surface, meanwhile, the main failure modes were fiber tension fracture, fiber pull-out and interface debonding.

Of all impacted specimens, one constituent, the BMI-A resin, changed color to light yellow around the damage area on both surfaces. On the back surface, the color change was along the weft yarns and warp yarns, which also coincided with the fiber tension fracture failure mode.

Generally, the composite damage was quite localized, which showed the 3DOWCs have superior resistance to crack initiation and propagation. With high speed camera recording, it was observed that under impact loading lower or equal to the ballistic limit, the composite target underwent large bending deflection. It fluctuated back and forth for several times, which dissipated most of the kinetic energy. The energy absorbed due to deformation was the one of the most important energy absorption mechanism.

**Figure 6:**
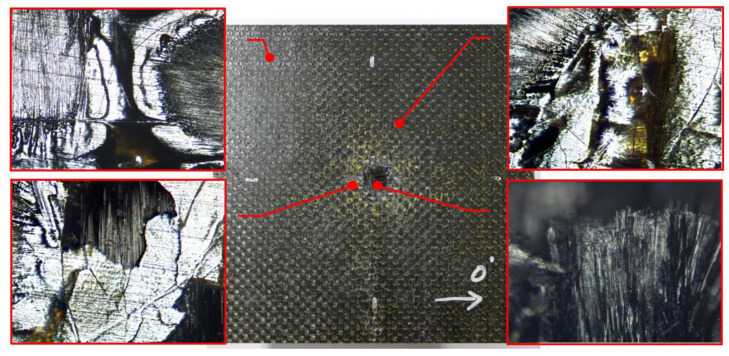
Composites surface damage on the front surface in test 11.

Microscope photos of composite surface in test 11 were shown in Figure 6. At the border section that was far away from the impact center, there were no obvious defects and damage. As it got closer to the center damage area, the matrix showed cracks and abscission, which led to very weak interface adhesion. But the fibers, at least on the surface, were still tough and seldom showed breakage. Not until reaching the impact center, the fiber breakage was seen where the projectile left its trail as a rounded hole.

## 3. Finite Element Simulation

It is well known that a thorough and complete experimental investigation of the behavior for textile composites under impact loading is impractical for its high cost, labor intensity and time taken. Thus, the finite element method is considered to be the cost-effective alternative to have a preliminary understanding of the behavior of composites with complicated structure. In this section, we explain how a multiscale modeling method was performed from microscale modeling to unit cell analysis and to macroscale simulation. Then a combined meso-macroscale modeling method is presented.

### 3.1. Micromchanical Modeling and Unit Cell Analysis

Fiber yarns could be considered as unidirectional composite with transversely isotropic material properties. Many micromechanical models are available to predict the effective elastic constants and strength—from the simple use of the rule of mixture based on a mechanics of material approach [26,27] to a complicated, detailed finite element method that accounts for the damage and failure behaviors of various constituents. The method of cells (MOC) micromechanics theory [28] is based on an idealized micromechanical arrangement, generally a doubly periodic RUC (Repeating Unit Cell). The code is available with MAC/GMC 4.0 [29], a micromechanical software which was developed at NASA Glen Research Center, which analyzes the thermoinelastic behavior of composite materials and laminates.

The overall fiber volume fracture can be verified by the material weight before and after resin injection, and by measuring the area of fiber tow cross section, it was accounted that the average fiber volume fraction in fiber tows were around 78%. Therefore, it was assumed that the average fiber volume fraction is 78% in all warp/weft/binder yarns. The predicted elastic constants and the strength of fiber yarns are shown in Table 4.

**Figure 7:**
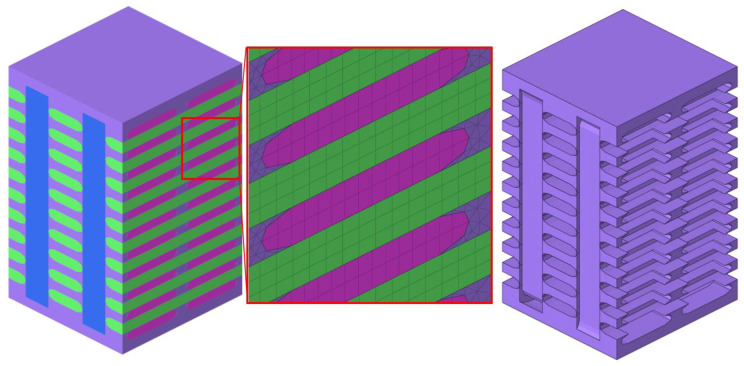
Unit cell geometry (**left**), detailed mesh (**middle**) and pure resin matrix (**right**).

The meso-modeling process began from the concept of a representative volume cell, known as unit cell [30]. It was the smallest portion of a composite whose behavior was presentative of the overall behavior of the entire composite. As shown in Figure 7, each individual constituent including the matrix (purple), the fiber tows in warp (green), weft (pink) and binder (blue) directions, was assembled to form the composite architecture. It was 5.264 mm in the warp direction, 5 mm in the weft direction and 8 mm in thickness, correlated with geometry parameters listed in Table 2. The eight-node solid hexahedron element (Hex 8, LS-DYNA), the six-node pentahedron element (Penta 6, LS-DYNA) and four-node tetrahedron element (Tetra 4, LS-DYNA) were used for the meshing. In the warp and weft fiber tows, all elements were Hex 8, while for the binder fiber tows, both Hex 8 and Penta 6 were involved. Because of the complex geometry of the matrix, all its elements were Tetra 4. There were overall 39,277 elements generated for a single unit cell. The bonding between constituents was modeled with the penalty-based contact *CONTACT_TIEBREAK_SURFACE_TO_SURFACE.

An orthotropic material with optional simplified damage and optional failure for composites could be defined. It was incorporated as Mat 221 [31] in LS-DYNA. Prior to damage initiation, the material model assumed linear elastic behavior. The progressive damage process could be realized in the model by applying a reduction to the three Young’s moduli and the three shear moduli. In addition, nine failure criteria on strains governed the failure of the element, at which state the material was said to have undergone complete failure, shown in Equation (1).

Tension fiber mode: $\varepsilon_{1t}\geq \varepsilon_{1tf}\left(\varepsilon_{1t}>0\right)$

Compressive fiber mode: $\;|\varepsilon_{1c}|\geq \varepsilon_{1cf}\left(\varepsilon_{1c}<0\right)$

(1) $$\begin{array}{ll} \mathrm{Tensile}\;\mathrm{matrix}\;\mathrm{mode}:\varepsilon_{2t}\geq \varepsilon_{2tf}(\varepsilon_{2t}>0);\;\varepsilon_{3t}\geq \varepsilon_{3tf}(\varepsilon_{3t}>0) & \end{array}$$

Compressive matrix mode: $\left|\varepsilon_{2c}\right|\geq \varepsilon_{2cf}(\varepsilon_{2c}<0)$; $\left|\varepsilon_{3c}\right|\geq \varepsilon_{3cf}(\varepsilon_{3c}<0)$

In-plane shear mode: $\left|\gamma_{12}\right|\geq \Gamma_{12f}$

Out-of-plane shear mode: $\left|\gamma_{13}\right|\geq \Gamma_{13f}$; $\left|\gamma_{23}\right|\geq \Gamma_{23f}$

When failure occurred, elements were deleted (erosion), and, under the control of the NERODE flat, failure may occur either when only a single integration point had failed, when several integration points had failed, or when all integrations points had failed.

**Figure 8:**
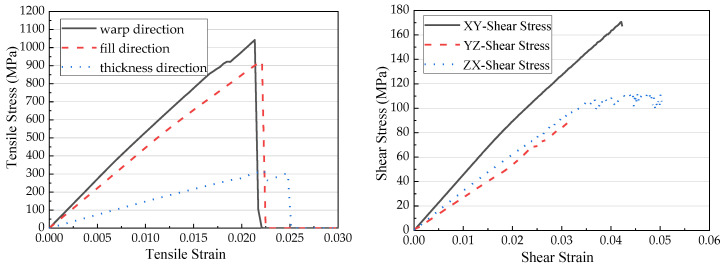
Numerical analysis results of unit cell.

It had been verified and validated by Harrington [32] that using virtual testing method can complete necessary characterization tests with finite element models. The system, here the unit cell, took in the material properties of the constituents and textile structures. Tensile virtual tests in the warp, weft and thickness direction and shear tests on xy, yz and zx plane were carried out. The load was displacement-controlled for each test. Results are plotted in Figure 8. This was effective to characterize the homogenized properties of a unit cell, and thus to have a rough understanding of the composites since the unit cell was the smallest repeating unit for the composites. 

### 3.2. Macroscale Modeling

**Figure 9:**
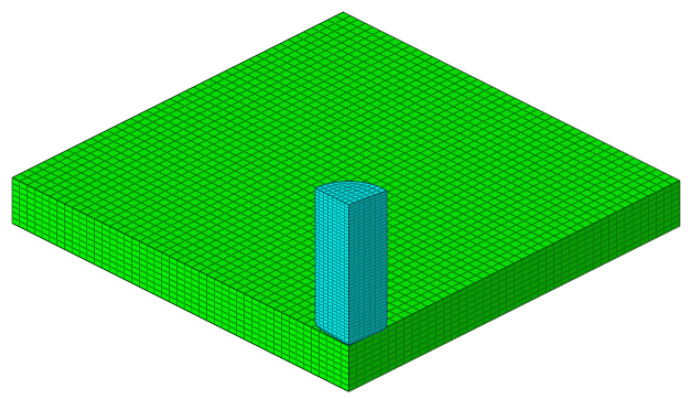
Numerical analysis results of unit cell (three-quarters of the model is hidden).

The macroscale represents the structural scale for analysis without considering the textile pattern of the composites. The finite element model used for numerical simulation analysis is shown in Figure 9. The projectile material TC4 used the Johnson–Cook model. Parameters were listed in Table 5, which referred to Liu. [33]. The projectile was modeled on the basis of the experimental schematic and was assigned with initial velocity. The composite panel was 150 mm × 150 mm × 8 mm. Four edges that were 5 mm in width were constrained in all translational and rotational directions. The projectile and the composites were both meshed with solid element, of which the size was around 1 mm, generating a total of 69,216 elements. The composite panel was assigned with material model Mat 221 in LS-DYNA. The model parameters were characterized by unit cell analysis, as listed in Table 6.

**Figure 10:**
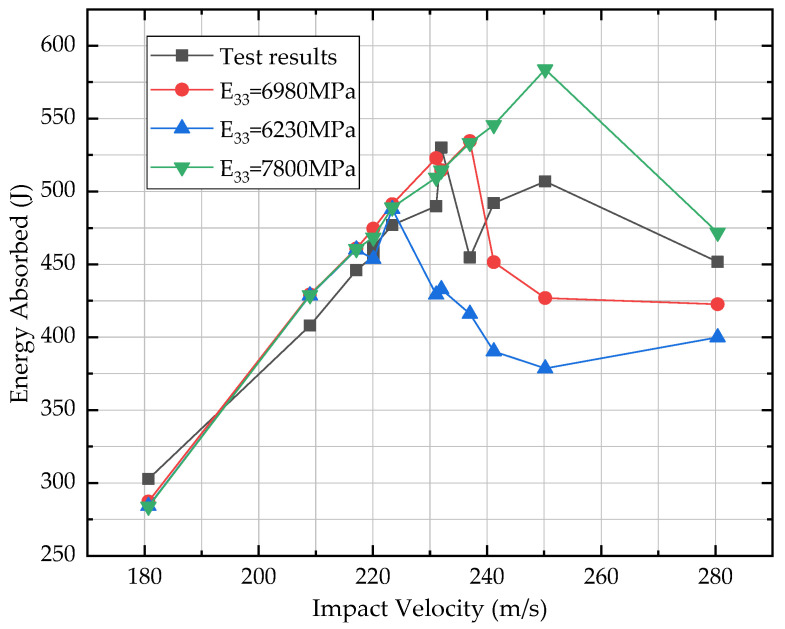
Simulated energy absorbed versus impact velocity.

Since the composite material model was characterized by a mesoscale unit cell analysis, it was confirmed that the homogenized mechanical constants prediction was close to real values in the warp and weft direction. For one thing, the warp and weft yarns were along the fiber’s longitudinal direction; and for another, the prediction was coincided well with published data [34,35]. So, the through thickness modulus E_33_ will be a varying parameter. Energy absorbed by the composites versus initial projectile velocity was plotted in Figure 10. It showed that larger E_33_ led to higher ballistic limit. When the composites were not penetrated and the projectile was rebounded back, the E_33_ seldom affected energy absorption. When projectile penetrated the composites, with the increase of E_33_, the composites absorbed more energy. Later analysis on the deformation and damage was based on E_33_ = 6,230 MPa. 

For the through thickness direction, it seems that the stronger it is, the better, according to the simulation result. However, it should be noticed that the measures to enhance the out-of-plane properties will weaken the in-plane performance as well. So, there should be a careful balance between the in-plane and out-of-plane properties from a design perspective. 

**Figure 11:**
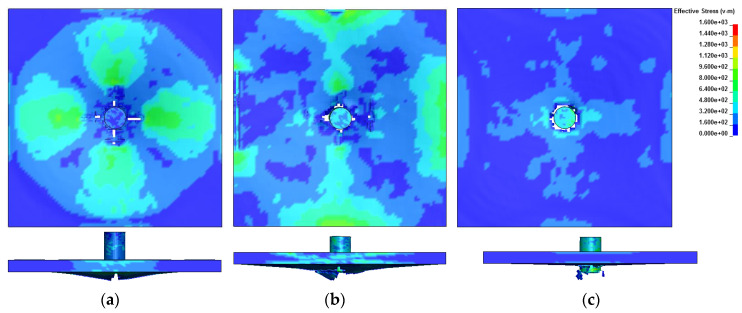
Composite damage after impact at (**a**) 209 m/s; (**b**) 237 m/s; (**c**) 280 m/s.

For the energy aspect, with a moderate E_33_, numerical simulation closely predicted the ballistic limit and the energy level when the impact velocity was lower than it. When velocity increased beyond that ballistic limit, there was an underestimation of energy that can be absorbed by composites, shown in Figure 10. Composites damage were shown in Figure 11 of the impact velocity 209, 237 and 280 m/s. In Figure 11a, large deformation was observed. A circular strain wave was evoked by the impact of projectile. Though the projectile was rebounded, cross-shaped damage and cracks were also generated on the back surface. In the case when penetration occurred, like in Figure 11b,c, the damage area changed from cross-shaped to circular with the increase of impact velocity. 

**Figure 12:**
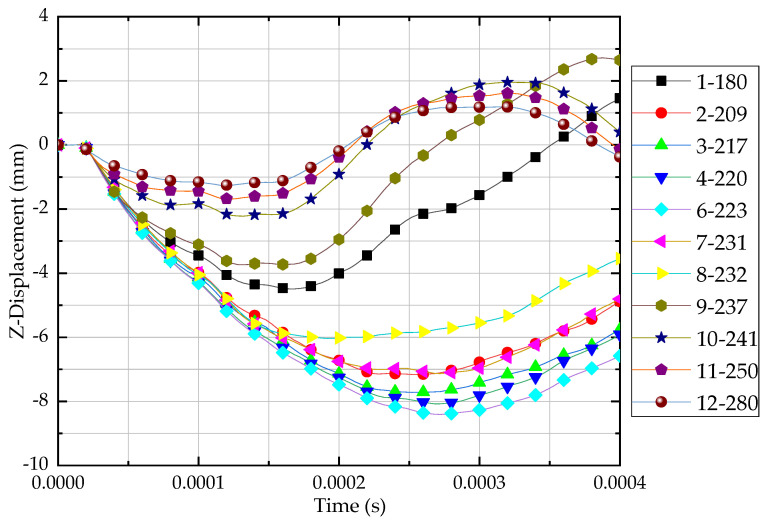
Out-of-plane deformation under different impact velocity.

Composite target deformation in the Z direction (also the out-of-plane direction) is plotted in Figure 12. With the increase of impact velocity, the z-displacement would increase first to a peak value around the ballistic limit and then decrease. For instance, when the impact velocity was 223 m/s, the out-of-plane displacement can be over 8 mm, larger than composites thickness. When the impact velocity was beyond 241 m/s, composites were perforated and the displacement magnitude was 1–2 mm, but under a higher frequency.

### 3.3. Combined Meso-Macroscale Modeling

It is very efficient with a macroscale model; however, the homogenized model was not sophisticated enough to characterize detailed local damage and discuss mesoscale topics such as yarn breakage, delamination, interface debonding and matrix cracks. At the mesomechanical scale, each unit cell consisted of over 39,000 elements. A mesoscale global model of the impacted composite panel would be so large to take much computing resources, like more than 31 million elements. In order for the combined meso-macroscale modeling approaches to be utilized in real applications for analyzing structure efficiently, methods and techniques were required to combine them into the same analysis model effectively and appropriately. It could be realized by the interface definitions for component analysis in LS-DYNA [36]. 

**Figure 13:**
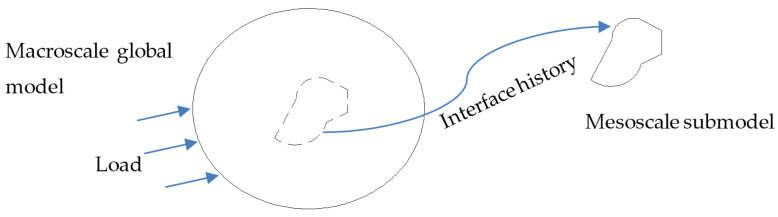
Illustration of the interface definition for component analysis.

Interface definitions for component analyses is one technique that could be used to study multiple levels of submodeling, which is a powerful tool for studying the detailed response of a small portion in a large structure. As shown in Figure 13, in the macroscale model, the user defined surfaces (dashed line) for which the displacement and velocity time histories were saved at a specified frequency. This data then would be used to drive interfaces in the mesoscale submodel. By doing so, the computing efficiency will be substantially higher. Composites, especially, have localized damage and failure are suitable for this multiscale modeling method.

The choice of interface location and local boundary requires some engineering judgement. The boundary of the submodel should be far away from the area where the response is changed by different modeling. In this study, the composite damage was quite localized from test observation. Considering the limited computing resources, 8 × 8 unit cells were chosen as the mesoscale submodel. The submodel was, therefore, 40 mm × 42 mm, with about 2.5 million elements, which was much less compared with the global mesoscale model of 31 million elements.

**Figure 14:**
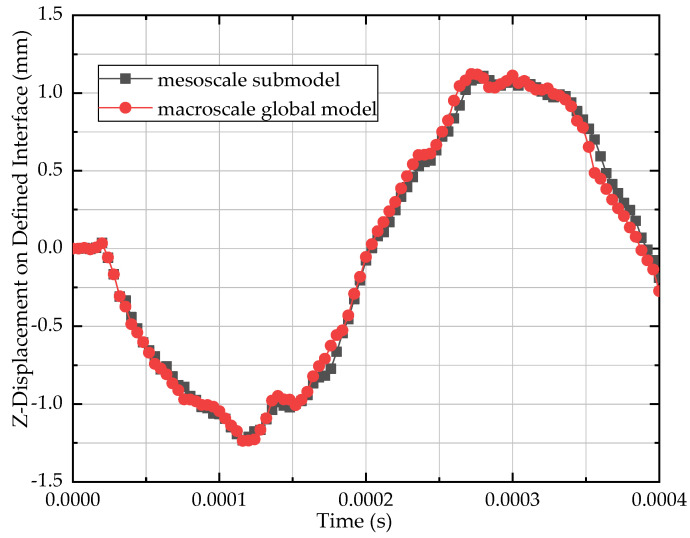
Comparison of out-of-plane displacement on the interface.

The effectiveness of the combined multiscale modeling method has been discussed by Nie [18] and Hu [20]. Here a simple verification was also shown in Figure 14. Very good consistency was obtained between the macroscale global model and the mesoscale submodel. 

**Figure 15:**
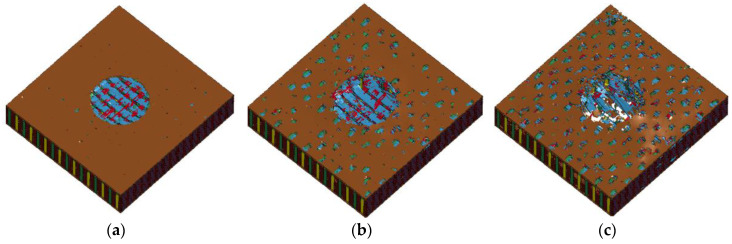

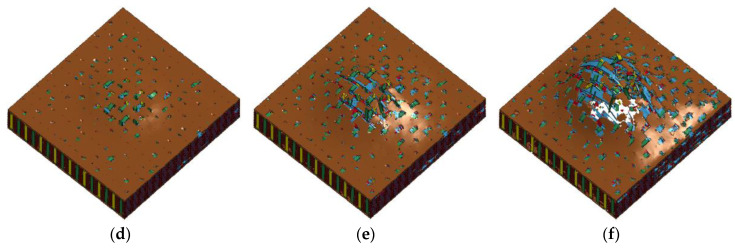
Composites damage of mesoscale modeling for (**a**–**c**) front surface and (**d**–**f**) back surface.

Composite damage of the front and back surfaces is shown in Figure 15. Detailed features such as delamination, fiber tow breakage and matrix crack were visible. In Figure 15a, it can be seen that the erosion of matrix formed a circle shape at the moment when the projectile came to impact the composites, and, as the projectile went further, the damage extended to the peripheral area and the matrix broke off from where it contacted with binder yarns. Fiber breakages were seen on both the front and back surfaces. On the front surface, the fiber yarns were under compression and shear. The breakage happened along the side of projectile. On the back surface, the fiber yarns were mainly under tension load, and the debris consisting of resin fragments and fiber yarns pieces flew out.

## 4. Conclusions

A series of impact tests was conducted regarding 3DOWCs. Impact velocity varied from 180 m/s to 280 m/s, amounting to an energy level of 320–774 J. Depending on the impact velocity, the projectile could rebound from, embed into or perforate the composite target. When impact velocity was 232 m/s, the projectile stuck in the composites. Therefore, the ballistic limit was around 232 m/s, which amounted to 530 J kinetic energy. The large deformation effect and the strain rate effect were both important for impact cases, especially when plane deflection was close to or exceeded its thickness. Composite damage was quite localized, which indicated that 3DOWCs have superior resistance to crack initiation and propagation. In the impact surface, the main failure modes were fiber shear failure and matrix crush, and in the exit surface, the main failure modes were fiber tension fracture, matrix cracking and delamination.

A multiscale modeling method was developed to investigate the impact resistance of 3DOWCs. Microscale analysis was used to characterize unidirectional fiber/resin properties, including elastic constants and strength. The mesoscale model was capable of considering the detailed woven geometry and architecture as well as the mechanical behavior of fiber tows and matrix. Then, a combined multiscale modeling method, which enabled the full use of advantages of both the macroscale and mesoscale model, was proposed. The prediction of the ballistic limit was sensitive to out-of-plane properties. Higher E_33_ led to higher critical ballistic limit. When the projectile rebounded, the out-of-plane deformation could reach to 8 mm; when the composites was penetrated, the deformation decreased to a lower level, and the composites vibrated with a higher frequency. With the increase of impact velocity, the damage area transformed from a cross shape to a circular hole. This phenomenon was also observed from tests where the resin color changed along the warp and weft directions. A mesoscale submodeling analysis provided more detailed observations, such as delamination, fiber yarn breakage and matrix cracks, and it has been proved that the combined multiscale modeling improved both efficiency and accuracy for explicit dynamic analysis.

## Figures and Tables

**Table 1:** Properties of composite components.

Property ^1^	Fiber	Matrix
Material type	Toray T700	BMI-A resin
Density (g/cm^3^)	1.8	1.27
Axial modulus (GPa)	230	4.03
Transverse modulus (GPa)	15	4.03
Shear modulus (GPa)	24 (Axial)/5.03 (Transverse)	101
Tensile strength (MPa)	4900	107.1
Shear strength (MPa)	-	100.7
Filament diameter (μm)	7	-
Glass transition temperatures (°C)	-	~250

^1^ Fiber properties were from Toray technical data sheet. Matrix properties were from lab.

**Table 2:** Structural parameters of composites.

Parameters	Composites Studied	Parameters	Composites Studied
Textile type	3D orthogonal woven	Density in weft (yarns/cm)	3.8
Fibers per yarn	12k	Thickness (mm)	~8
Density in warp (yarns/cm)	4	Fiber volume fraction (%)	~54

**Table 3:** Summary of ballistic impact tests.

Test No.	Projectile Velocity (*Vi*, m/s)	Initial Kinetic Energy (J)	Residual Velocity ^1^ (*Vr*, m/s)	Absorbed Energy (J)
1	180.7	319.99	−41.9	302.79
2	209.0	430.26	−47.5	408.03
3	217.1	461.90	−40.3	445.98
4	220.1	474.75	−44.1	455.69
5	220.1	474.75	−35.9	462.12
6	223.4	491.59	−38.5	476.99
7	231.1	523.39	−58.4	489.97
8	232.0	530.17	0	530.17
9	237.0	553.26	100	454.76
10	241.2	570.14	89.3	491.99
11	250.2	613.48	104.3	506.87
12	280.4	774.45	181	451.75

^1^ Residual velocities of negative values indicated that the projectile was rebounded back and the positive values indicate penetration.

**Table 4:** Predicted properties of fiber yarns.

Property ^1^	Fiber Yarn
Elastic Modulus $E_{11}$, MPa	180,287
Elastic modulus $E_{22}$, MPa	11,381
Poisson ratio,$\;v_{12}$	0.24356
Shear modulus $G_{12}$, MPa	7863
Shear modulus $G_{23}$, MPa	3962
Tension strength $F_{1T}$, MPa	3841
Tension strength $F_{2T}$, MPa	356
Compression strength $F_{1C}$, MPa	1538
Compression strength $F_{2C}$, MPa	422
Shear strength $F_{S}$, MPa	2302

^1^ Fiber direction was denoted as “1” and the transverse plane as “2–3”.

**Table 5:** Johnson–Cook model parameters for projectile material TC4.

***E* (GPa)**	**ν**	***ρ* (kg/m^3^)**	***T*_m_ (K)**	***T*_r_ (K)**	*C*_p_ (J/kg*K)	$\dot{\varepsilon_{0}}$ **(s^−1^)**	***A* (MPa)**	***B* (MPa)**
113	0.33	4.43 × 10^3^	1878	293	580	1	1089	1083
***C***	**n**	**m**	***D*_1_**	***D*_2_**	***D*_3_**	***D*_4_**	***D*_5_**	
0.014	0.93	1.1	−0.09	0.27	0.48	0.014	3.87	

**Table 6:** Mat 221 model parameters for macroscale modeling.

***ρ* (kg/m3)**	***E*_11_ (GPa) 1**	***E*_22_ (GPa)**	***E*_33_ (GPa)**	***v*_21_**	***v*_32_**
1.563 × 10−9	55.4	50.21	6.98	0.1618	0.1535
***G*_12_ (MPa)**	***G*_23_ (MPa)**	***G*_31_ (MPa)**	**$\varepsilon_{1tf}$**	**$\varepsilon_{2tf}$**	$\varepsilon_{3tf}$
3580	2220	2440	0.021	0.021	0.025

^1^ Warp direction was denoted as “1”, weft direction as “2” and thickness direction as “3”.

## References

[1] Saleh M.N., Soutis C. (2017). Recent advancements in mechanical characterization of 3D woven composites. Mech. Adv. Mater. Mod. Process..

[2] Goering J., McClain M. Recent Developments In 3D Woven Pi Preforms. Proceedings of the American Society for Composites, 22nd Technical Conference.

[3] Gardiner G. (2014). 3D Preformed Composites: The Leap into LEAP. https://www.compositesworld.com/articles/3-d-preformed-composites-the-leap-into-leap.

[4] (2005). CCAR-33, Airworthiness Standards: Aircraft Engines.

[5] Potluri P., Hogg P., Arshad M. (2012). Influence of fibre architecture on impact damage tolerance in 3D woven composites. Appl. Compos. Mater..

[6] Bahei-El-Din Y.A., Zikry M.A. (2003). Impact-induced deformation fields in 2D and 3D woven composites. Compos. Sci. Technol..

[7] Luo Y. (2007). Transverse impact behavior and energy absorption of three-dimensional orthogonal hybrid woven composites. Compos. Struct..

[8] Lv L., Sun B., Qiu Y., Gu B. (2006). Energy absorptions and failure modes of 3D orthogonal hybrid woven composite struck by flat-ended rod. Polym. Compos..

[9] Hao A., Sun B., Qiu Y. (2008). Dynamic properties of 3-D orthogonal woven composite T-beam under transverse impact. Compos. A Appl. Sci. Manuf..

[10] Tsai P., Jeng Y., Lee J., Stachiv I., Pittner P. (2017). Effects of carbon nanotube reinforcement and grain size refinement mechanical properties and wear behaviors of carbon nanotube/copper composites. Diam. Relat. Mater..

[11] Xuan H., Liu L., Chen G. (2013). Impact Response and damage evolution of triaxial braided carbon/epoxy composites. Part I: Ballistic impact testing. J. Text. Res..

[12] Cater C.R., Xiao X., Goldberg R.K., Kohlman L.W. (2015). Single ply and multi-ply braided composite response predictions using modified subcell approach. J. Aerosp. Eng..

[13] Antin K.N., Laukkanen A., Andersson T., Smyl D., Vilaca P. (2019). A multiscale modeling approach for estimating the effect of defects in unidirectional carbon fiber reinforced polymer composites. Materials.

[14] Ivanov D.S., Baudry F., Broucke B.V.D., Lomov S.V., Xie H., Verpoest I. (2009). Failure analysis of triaxial braided composite. Compos. Sci. Technol..

[15] Littell J.D., Binienda W.K., Roberts G.D., Goldbert R.K. (2009). Characterization of damage in triaxial braided compos-ites under tensile loading. J. Aerosp. Eng..

[16] Kohlman L.W., Bail J.L., Roberts G.D., Salem J.A., Martin R.E., Binienda W.K. (2012). A notched coupon approach for tensile testing of braided composites. Compos. Part A Appl. Sci. Manuf..

[17] Li X., Binienda W.K. (2010). Mesomechanical Model for Numerical Study of Two-Dimensional Triaxially Braided Composite. J. Eng. Mech..

[18] Hu Y., Binienda W.K. Combined multiscale modeling of two-dimensional triaxially braided composites. Proceedings of the Internationals Symposium on Structural Integrity.

[19] Nie Z. (2014). Advanced Mesomechanical Modeling of Triaxially Braided Composites for Dynamic Impact Analysis with Fialure. Ph.D. Thesis.

[20] Sun M. (2018). Multiscale Hybrid Element Modeling of Triaxial Braided Composite.

[21] Iredale R.J., Ward C., Hamerton I. (2017). Modern advances in bismaleimide resin technology: A 21st century perspective on the chemistry of addition polyimides. Prog. Polym. Sci..

[22] Calladine C.R., Heyman J., Lechie F.A. (1968). Simple ideas in the large-deflection plastic theory of plates and slabs. Engineering Plasticity.

[23] Yu T., Qiu X. (2011). Impact Dynamics.

[24] Ma P., Jin L., Wu L. (2019). Experimental and numerical comparisons of ballistic impact behaviors between 3D angle-interlock woven fabric and its reinforced composite. J. Ind. Text..

[25] Li Z., Sun B., Gu B. (2010). FEM simulation of 3D angle-interlock woven composite under ballistic impact from unit cell approach. Comput. Mater. Sci..

[26] Chamis C.C. Simplified composite micromechanics equations for strength, fracture toughness, and environmental effects. Proceedings of the 29th Annual Conference of the Society of the Plastics Industry.

[27] Chamis C.C. (1989). Mechanics of composite materials: Past, present and future. J. Compos. Tech. Res..

[28] Aboudi J., Arnold S.M., Bednarcyk B.A. (2013). Micromechanics of Composite Materials: A Generalized Multiscale Analysis Approach.

[29] Bednarcyk B.A., Arnold S.M. (2002). MAC/GMC 4.0 User’s Manuals-Keywords Manual, NASA/TM-2002-212-77/VOL2.

[30] Mishnaevsky L., Schmauder S. (2001). Continuum mesomechanical finite element modeling in materials development: A state-of-the-art review. Appl. Mech. Rev..

[31] (2019). LS-DYNA Keyword User’s Manual, Volume II Material Models.

[32] Harrington J. (2015). Using Virtual Testing for Characterization of Composite Materials. Master’s Thesis.

[33] Liu L. (2013). Research on the Containment of 2D Carbon Fiber Triaxial Braided Tape Wound Composite Casing. Ph.D. Thesis.

[34] Lomov S.V., Bogdanovich A.E., Karahan M., Mungalov D., Verpoest I. Mechanical behaviour of non-crimp 3D woven carbon/epoxy composite under in-plane tensile loading. Proceedings of the 18th International Conference of Composite Materials.

[35] Bogdanovich A.E., Karahan M., Lomov S.V., Verpoest I. (2013). Quasi-static tensile behavior and damage of carbon/epoxy composite reinforced with 3D non-crimp orthogonal woven fabric. Mech. Mater..

[36] (2019). LS-DYNA Keyword User’s Manual, Volume I.

